# The Rationale of Influenza

**Published:** 1891-01

**Authors:** 


### THE RATIONALE OF INFLUENZA.

---

The following remarks by Dr. Laffont, Professor de Thèrapeutique à la Faculté de Mèdecine de Lille, will be read with interest: The epidemic which was such a cruel scourge last winter is again appearing, although up to the present in a milder form. It may, therefore, not be without use to consider at the present moment the most rational treatment of this affection, at all times painful, and sometimes, from its complications, serious. This malady is, I consider, a contagious catarrhal affection, in its milder form known to us as "grippe," but from its recent serious epidemic character christened "influenza, " a name it will probably retain henceforth. The symptoms of this complaint are manifested invariably by a functional depression, more or less marked, of the whole system, varying from simple lassitude, stuffiness of the nose and slight gastric obstruction, all premonitory symptoms of a large number of contagious diseases, and fortunately often constituting the only symptoms of the malady, which in such cases passes for ordinary "grippe."

In the late epidemic, to these premonitory symptoms succeeded all the characteristics of grave typhoid infection: nausea, fever, muscular pains, delirium, pneumonia, with tendency to suffocation and complete prostration. In the discussions at societies and in medical journals on its etiology, some described it as a simple catarrhal affection, more or less grave, having for cause the influence of the external conditions of the atmosphere, and denied its contagious character, others sought at once for the microbe. In the midst of these etiological discussions, no therapeutic law was propounded, and the medical journals were advocating here aperient medicine, antithermics; there, the vin mariani (made from the coca of Peru) and tonic medicines; elsewhere, counter-irritation and balsamics were said to do wonders ; almost everywhere was admitted the specific effect of sulphate of quinine, or still better, salts of quinine, above all, antipyrin. From my own experience, based upon a great number of cases and on myself in particular, I have no hesitation to assert that the method which succeeded the best was essentially eclectic. Thus, at its first manifestation I was able to arrest the development of the disease by administering an aperient (oleum ricini by preference), then causing thoracic revulsion by rubefaction, or even vesication, and by provoking simultaneously a non-depressing diaphoresis, easely obtained by administering several times in the day a grog made from vin mariani, one-third wine and two-thirds water, very hot, with sugar, such as has been prescribed by the learned laryngologist, Fauvel, for hoarseness and loss of voice, "a frigore."

In the presence of influenza in the stage when the patient was completely depressed, very far from ordering antipyrin, which only augments the depression, I found it much more effectual to administer strong tonics, such as generous wines, champagne, whiskey, rum, cognac, tonics, physical and moral, such as the preparations of coca mariani, vin and elixir, at the same time causing revulsion, and administering repeated aperients. From this treatment I rapidly cured myself, and observed the same results in patients without that long and tedious convalescence due, as I think, to the weakness caused by the use of antipyrin.

I advise, then, as a rational treatment for influenza and kindred affections: first, gentle purgatives; second, diaphoretics and revulsives; third, strong tonics.—*The Medical Press and Circular. London, November 19, 1890.*

